# Diagnosis and treatment in adult patients with C3 glomerulopathy in Japan: a real-world survey

**DOI:** 10.1007/s10157-025-02779-5

**Published:** 2025-11-03

**Authors:** Naoki Nakagawa, Yutaro Kotobuki, Michel Kroes, Shunsuke Eguchi, Toshinaga Tsuji, Alice Simons, Susanna Libby, Raisa Sidhu, Serge Smeets, Kazuma Iekushi

**Affiliations:** 1 Division of Cardiology and Nephrology, Department of Internal Medicine, Asahikawa Medical University, Asahikawa, Japan; 2 Novartis Pharma K.K., Tokyo, Japan; 3 Novartis Pharma AG, Basel, Switzerland; 4 Adelphi Real World, Bollington, UK

**Keywords:** Glomerulonephritis, Kidney disease, C3 glomerulopathy

## Abstract

**Introduction:**

C3 glomerulopathy (C3G) is an ultra-rare, complex, under-recognized kidney disease with a challenging diagnosis and no approved treatment. Nephrologists were surveyed to understand the treatment and management of C3G. This study presents the diagnostic challenges and treatment patterns of Japanese patients with C3G.

**Methods:**

Data from the Adelphi C3G Disease Specific Programme™, a multinational survey of nephrologists treating patients with C3G in 8 countries including Japan, were retrospectively analyzed. Nephrologists completed patient-record forms on patient demographics, diagnosis, clinical characteristics, and treatment approaches.

**Results:**

Sixteen nephrologists from Japan responded to the survey for 36 patients with C3G. Mean age at diagnosis and at the time of the survey was 45.4 and 48.6 years, respectively. Common symptoms at diagnosis were proteinuria (100%) and hematuria (83%); 79% of patients had proteinuria of  ≥1 g/day, and 3% had an estimated glomerular filtration rate of <30 mL/min/1.73 m^2^. Median time from initial examination by general practitioner to definitive diagnosis was 8.4 weeks; ~20% and 10% of patients had to wait for >4 and >8 months, respectively, to get a confirmed C3G diagnosis; and 69% of patients had an additional biopsy. Angiotensin receptor blockers (68%), corticosteroids (64%), and sodium-glucose cotransporter-2 inhibitors (25%) were the main treatments utilized. Physicians perceived 19% of patients to have a gradually deteriorating disease condition.

**Conclusion:**

This survey analysis explored the current status in diagnosis and management of patients with C3G in Japan. The lack of specific treatments emphasizes the need for novel targeted therapies addressing the root cause of C3G.

**Supplementary Information:**

The online version contains supplementary material available at 10.1007/s10157-025-02779-5.

## Introduction

C3 glomerulopathy (C3G) is an ultra-rare kidney disease caused by overactivation of the alternative complement pathway (AP), progressing to kidney failure in ~50% of patients within 10 years of diagnosis [1, 2]. It is a type of membranoproliferative glomerulonephritis (MPGN) characterized by deposition of C3 in the glomerulus [2, 3].

C3G is further divided into 2 major subtypes: C3 glomerulonephritis (C3GN) and dense deposit disease (DDD), which can be distinguished by differences in the density and location of C3 deposits, as observed through electron microscopy [3–5]. The worldwide annual incidence of C3G is estimated to be 1 to 2 cases per million [6], though its incidence in Japan is currently unknown. C3G is known to affect individuals of all ages, with many of them experiencing symptoms early in childhood and young adulthood [2, 7]. When patients reach the stage of kidney failure, undergoing dialysis or transplantation is necessary. However, there is a significant risk of C3G disease recurrence [8].

One of the challenges in diagnosing C3G is its heterogeneous clinical presentation, making it difficult to distinguish it from other kidney disorders [9]. The range of symptoms in C3G is broad and commonly includes hematuria, proteinuria, and reduced kidney function, as determined by estimated glomerular filtration rate (eGFR) [10, 11]. To accurately diagnose C3G, a kidney biopsy and expert interpretation of its findings are crucial [6, 9].

Currently, there are no specific treatment options available that target C3G directly. The standard of care (SoC) for C3G, as outlined by the 2021 Kidney Diseases Improving Global Outcomes (KDIGO) guidelines, includes supportive measures such as angiotensin II receptor blockers (ARB) or angiotensin-converting enzyme inhibitors (ACEi) and immunosuppressive agents such as mycophenolate mofetil (MMF) and corticosteroids (CS) [10]. These treatment approaches, however, are non-targeted and lack robust evidence from clinical trials to support their use in patients with C3G [12, 13].

The overactivation of the alternative pathway (AP) is central to C3G pathogenesis, leading to the deposition of C3 and its cleavage products in the glomeruli. Therefore, it is crucial to inhibit the AP to prevent kidney inflammation and glomerular damage. This emphasizes the requirement for a treatment approach that specifically targets the underlying mechanisms of C3G [14].

The demographics, clinical and laboratory characteristics, and current treatment options available for patients with C3G in routine clinical practice in Japan were previously reported concurrently with IC-MPGN in a multicenter cohort study using the data from the Japan Renal Biopsy Registry [4]. There is a scarcity of evidence for C3G in Japanese population, and therefore, it is important to report as much evidence as possible for the diagnosis and treatment of Japanese patients with C3G.

While the previous real-world study on patients with C3G derived its outcomes from national biopsy registry, the present article shows the data from a real-world multinational survey, including Japan, captured by nephrologists treating patients with C3G. The purpose of this study was to gain an in-depth understanding of how patients with C3G are diagnosed and managed. The objective of our analysis is to describe the demographics, time to diagnosis, current patient journey, clinical characteristics, disease severity at diagnosis, and treatment approaches of patients with C3G in Japan.

## Methods

### Study design and participants

This was a retrospective analysis of data obtained from the Adelphi C3G Disease Specific Programme™ (DSP) through a cross-sectional multinational survey of 129 C3G-treating nephrologists in 8 countries (EU5 [France, Germany, Italy, Spain, United Kingdom], United States of America [USA], Japan, and China) between June 2022 and April 2023. More details on DSP methodology have been reported and validated previously [15–17].

During the data collection phase of the DSP, nephrologists were recruited in each country. They were compensated to participate in the DSP according to fair market rates, consistent with the time involved. Nephrologists actively managing patients with C3G (defined as seeing at least one eligible patient in a typical 3-month period) and responsible for treatment decisions were eligible for participation in the survey, which was designed to capture their perceptions and attitudes toward the management of C3G.

After an initial examination of patients, nephrologists completed structured patient-record forms for eligible patients with a confirmed C3G diagnosis. These forms captured comprehensive information such as demographics, clinical characteristics, patient diagnosis journey (including time to definitive diagnosis and reason for delay in diagnosis), treatment patterns (including prescription tendency), and treatment effect from the viewpoint of nephrologists. A questionnaire survey conducted retrospectively on the patient’s condition included information on severity, proteinuria, eGFR, and symptoms at diagnosis.

The questionnaires were anonymized, and an assigned physician number allowed for linking results between physician and respective patients.

## Assessments

The parameters assessed were patient demographics such as age, sex, and body mass index (BMI), time to diagnosis, current patient journey, clinical characteristics at diagnosis with respect to disease severity, and treatment approaches. Clinical characteristics at diagnosis and time of the survey were evaluated and stratified by degree of proteinuria (<1 g/day and ≥1 g/day).

All analyses were descriptive, and continuous variables were summarized by mean (standard deviation [SD]) or median (interquartile range [IQR]). Categorical variables were summarized by counts and percentages. The detailed methodology has also been reported previously [18].

## Results

### Questionnaire survey on C3G

Overall, 129 nephrologists from 8 countries completed record for 385 patients with C3G. Of these, 16 nephrologists from Japan responded to the questionnaire survey for 36 patients. Referrals from general practitioners accounted for 54%, and nephrologists consulted 5.4 patients with C3G on average over 3 months.

### Patient demographics and clinical characteristics

Physician-reported patient demographics and clinical characteristics of Japanese patients with C3G at diagnosis and at the time of the survey are summarized in Table 1. The mean (SD) age at the time of survey was 48.6 (12.4) years and at diagnosis was 45.4 (13.3) years, and 67% of patients were male. All the patients included at the time of the survey were ≥18 years of age. The majority of the patients (97%) had C3GN and only one patient had the DDD subtype. Regarding perceived disease severity, 24% of physicians reported their patients having mild C3G, and 55% and 21% reported their patients having moderate and severe C3G, respectively.

**Table 1:** Physician-reported patient demographics and clinical characteristics of patients with C3G

Parameter	*N*	
Age at diagnosis		
Mean (SD)	36	45.4 (13.26)
Median		48.5
Age at the time of survey		
Mean (SD)	36	48.6 (12.38)
Median		51.5
Sex, *n* (%)		
Male	36	24 (67)
Female		12 (33)
Body mass index, kg/m^2^		
Mean (SD)	36	21.1 (2.77)
C3G subtype, *n* (%)	34	
C3G		33 (97)
DDD		1 (3)
Severity at diagnosis, *n* (%)	33	
Mild		8 (24)
Moderate		18 (55)
Severe		7 (21)
Disease characteristics	29	
Proteinuria at diagnosis, g/day		
Median (IQR)		1.5 (1.0, 3.0)
eGFR at diagnosis, mL/min/1.73 m^2^		
Median (IQR)		50.0 (35.0, 75.0)
CKD stage at diagnosis (eGFR), (%)		
Stage 1 (>90)		13%
Stage 2 (60–89)		26%
Stage 3a (45–59)		29%
Stage 3b (30–44)		29%
Stage 4 (15–29)		3%
Stage 5 (<15)		0%
UPCR, ≥1 g/day, *n* (%)		23 (79)
Kidney transplantation, *n* (%)		0 (0)

*C3G* C3 glomerulopathy, *CKD* Chronic kidney disease, *DDD* Dense deposit disease, *eGFR* Estimated glomerular filtration rate, *IQR* Interquartile range, *SD* Standard deviation, *UPCR* Urine protein–creatinine ratio

The most common signs and symptoms reported by a physician at diagnosis were proteinuria (100%), hematuria (83%), edema (36%), hypertension (28%), and dyslipidemia (19%). As reported by physicians, 79% of patients had proteinuria of  ≥1 g/day, and 3% of patients had eGFR <30 mL/min/1.73 m^2^ at diagnosis. None of the patients ever received a kidney transplant. At diagnosis, the median (IQR) total protein to creatinine ratio was 1.5 (1.0–3.0) g/day, and the median (IQR) eGFR was 50.0 (35.0–75.0) mL/min/1.73 m^2^.

### Diagnosis journey

**Fig. 1:**
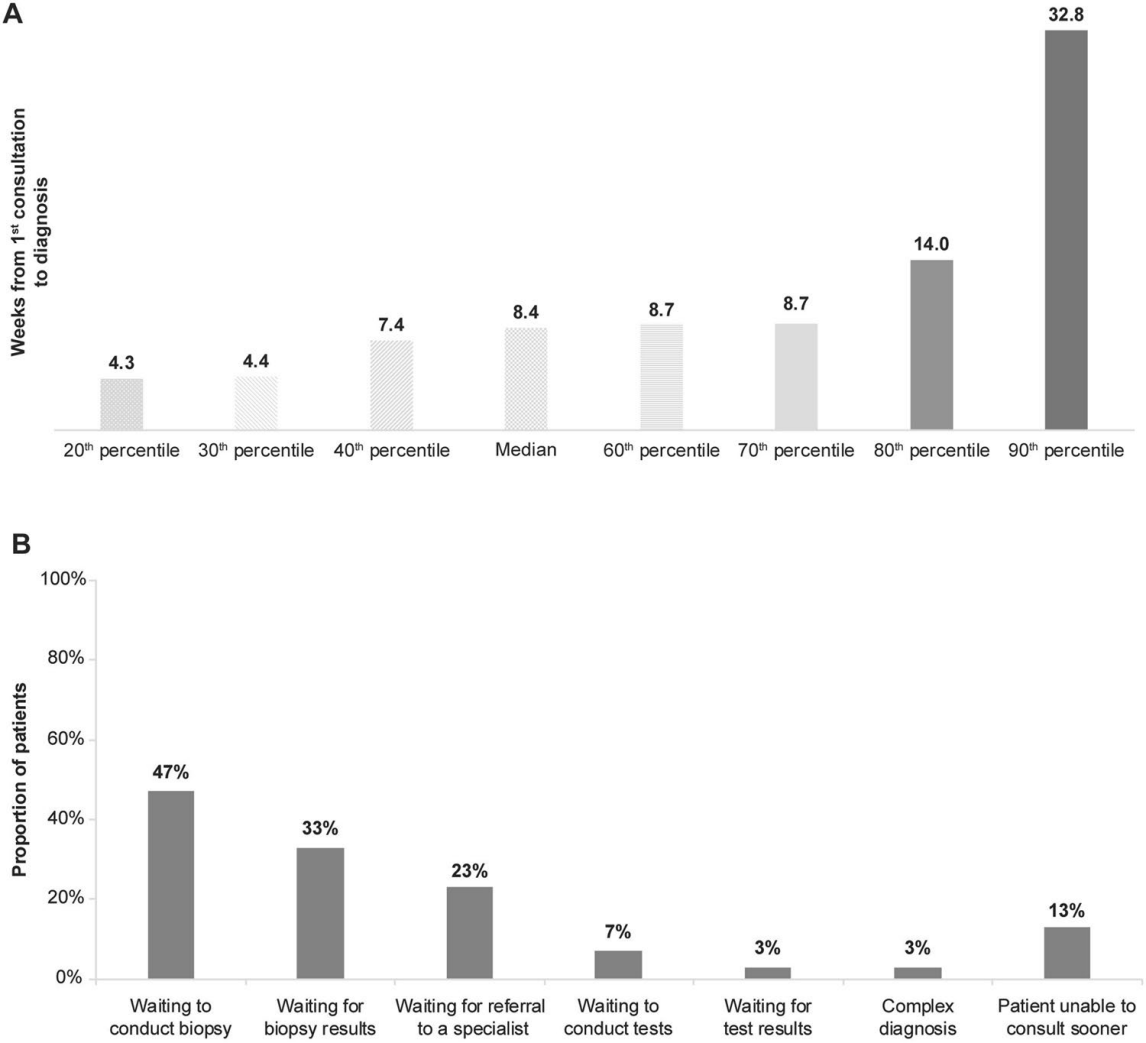
(**A**) Time from first physician visit to diagnosis (**B**) Physician-reported reasons for delay between first examination at GP to diagnosis (weeks)

The median time from the initial examination to definitive diagnosis was 8.4 weeks (Fig. 1A), and approximately 20% of patients had to wait for >14 weeks, and 10% had to wait for >32 weeks to get a confirmed C3G diagnosis. The median time between diagnosis and the time of the survey was 31.9 months. The major reasons for delay between initial examination and diagnosis reported by physicians were waiting to conduct a biopsy (47%), waiting for biopsy results (33%), and waiting for a referral to a specialist (23%) (Fig. 1B).

Of the 36 patients with C3G, 31% had no additional biopsy, while 69% had one additional biopsy. The top 3 reasons for additional biopsy were reported to be significant proteinuria (72%), hematuria (36%), and rapidly declining eGFR (16%).

### Treatment pattern at the time of survey

**Fig. 2:**
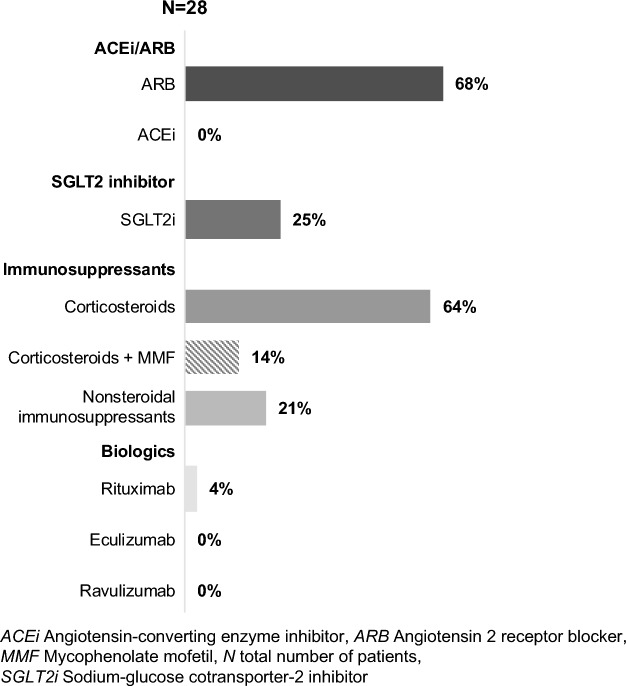
Physician-reported treatment received by the patients at the time of survey

As reported by physicians, the treatments that patients received at the time of survey were ARBs (68%), sodium-glucose cotransporter-2 inhibitors (SGLT2i; 25%), corticosteroids (CS; 64%), CS/MMF (14%), and rituximab (4%) (Fig. 2).

### Therapeutic effect from the physician’s perspective

**Fig. 3:**
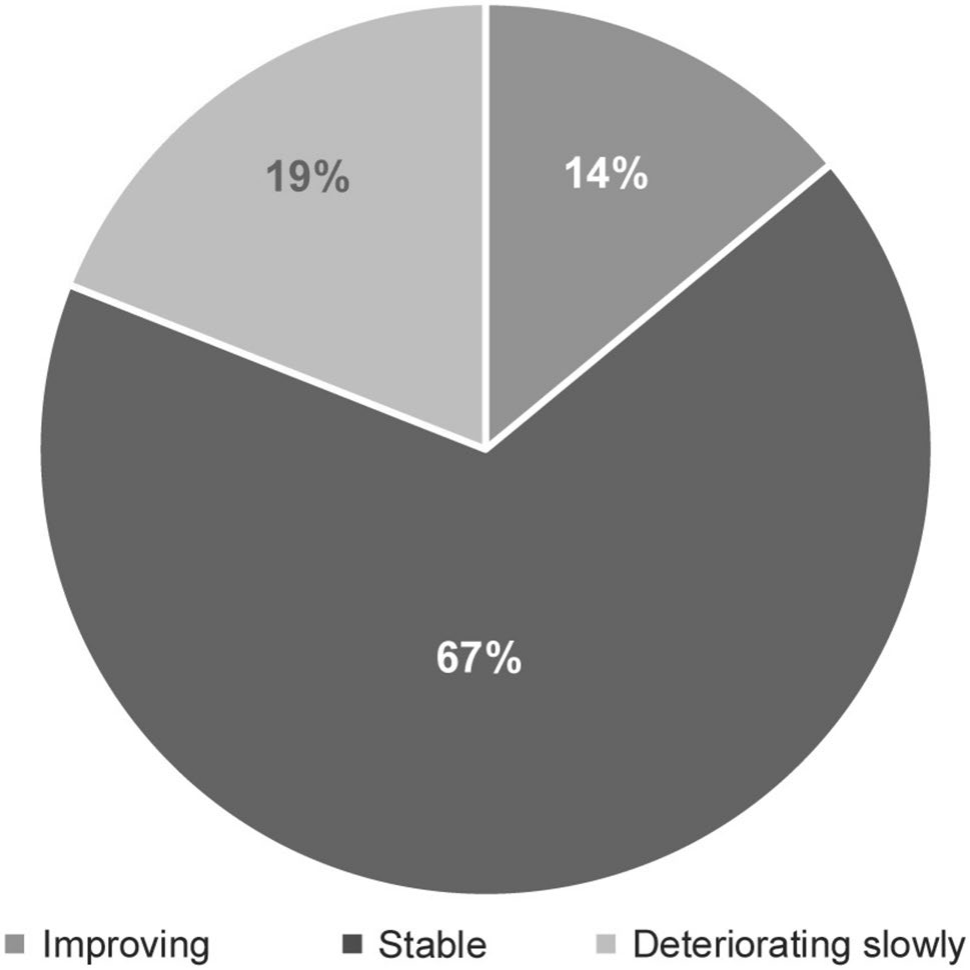
Physician-reported patient condition at the point of survey (*n* = 36)

**Fig. 4:**
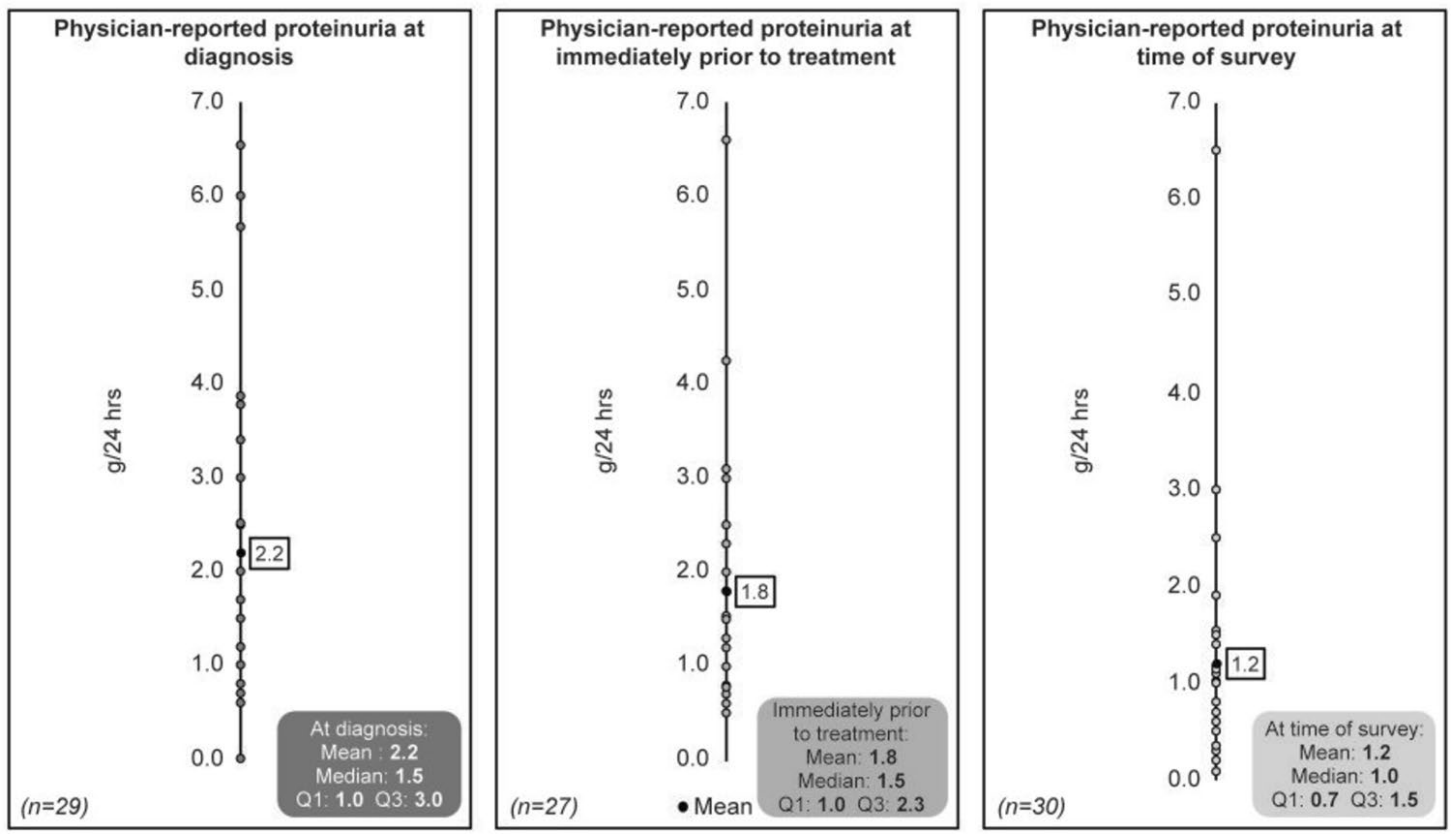
Physician-reported proteinuria over time

With regard to physicians’ perception on patient condition at the time of survey, the majority of the patients (67%) were reported to be in a stable condition, 14% of patients showed improvement, 19% of patients showed slow deterioration, and no patient showed rapid deterioration (Fig. 3). The mean proteinuria at diagnosis of C3G was 2.2 g/day (median 1.5; *n* = 29), immediately prior to the treatment was 1.8 g/day (median 1.5; *n* = 27) and at the time of survey was 1.2 g/day (median 1.0; *n* = 30) (Fig. 4).

## Discussion

This was a retrospective analysis of the Japanese population from a multinational real-world study to evaluate the disease burden and management of patients with C3G. The outcomes of the survey of 16 nephrologists for 36 patients with C3G they treated suggest that the majority of Japanese patients had moderate C3G, with 79% of patients experiencing high levels of proteinuria (≥1 g/day).

At the time of the survey, all the patients treated by nephrologists were aged ≥18 years, with a median age of 51.5, which is higher than that in another real-world study in Japan where patients had a median age of 19 years [4]. This could be because all the physicians who participated in the present survey from Japan were only nephrologists treating adult patients; none were treating pediatric patients.

The median time required from initial consultation to definitive diagnosis in patients with C3G was 8.4 weeks. It was observed that half of the Japanese patients waited for over 2 months for a confirmed C3G diagnosis as compared to the multi-country data where the median time from consultation to C3G diagnosis was 1.1 months [18]. Similarly, 10% of Japanese patients had to wait for over 8 months for C3G diagnosis in comparison to the multi-country data where the waiting time for 10% of patients was 5.0 months from initial consultation to C3G diagnosis. The longer waiting time for Japanese patients may be attributed to their potentially milder conditions, indicated by eGFR and proteinuria levels, which could be attributed to the annual health check-ups in Japan. This could be a potential reason for waiting longer to conduct a biopsy compared to patients in the multinational study. The major reasons for delayed diagnosis, as reported by the physicians from Japan, include waiting to conduct renal biopsy (47%), waiting for biopsy results (33%), and waiting for referral to a specialist (23%). These results are in line with the reasons reported in the multinational real-world survey, where 40% of nephrologists reported delay as they were waiting to conduct a biopsy, 32% reported waiting for biopsy results, and 30% reported to wait for referral to a specialist (Supplementary Fig. 1) [18]. Furthermore, majority of patients were reported to have one additional biopsy, mainly due to significant proteinuria, hematuria, and rapidly declining eGFR, although this survey did not collect data on the timing of the additional biopsy. Early diagnosis of C3G and quick commencement of treatment could be beneficial in slowing down disease progression and delaying the onset of kidney failure [19].

The most common symptoms observed at diagnosis in patients with C3G included proteinuria, hematuria, and edema, as observed in any chronic kidney disease (CKD). Physicians from other participating countries, such as the USA, the EU (France, Germany, Italy, Spain, and the UK), and China, also commonly reported these symptoms.

In comparison to the study by the Japan Renal Biopsy Registry, the eGFR reported in this study (50.0 mL/min/1.73 m^2^ vs 99.3 mL/min/1.73 m^2^) was lower, while proteinuria was higher (1.5 g/day vs 0.77 g/day) [4]. Compared to the multi-country data (*n* = 325) of this study, the median eGFR (*n* = 31) of Japanese population was almost the same (50.0 mL/min/1.73 m^2^ vs 49.0 mL/min/1.73 m^2^; Supplementary Fig. 2a), and the median proteinuria (*n* = 29) was lower (1.5 g/day vs 3.4 g/day; Supplementary Fig. 2b).

At the time of the survey, the median proteinuria remained at 1.0 g/day, despite patients receiving multiple lines of treatment. The Spanish group for the Study of Glomerular Diseases showed that time-averaged proteinuria below 1 g/day indicated a lower risk of kidney failure [20]. Given that proteinuria is an important prognostic marker of kidney decline, this suggests that more effective treatment options are needed for Japanese patients with C3G.

Physician-reported treatments included ARBs (68%), corticosteroids (64%), and SGLT2is (25%). Although the proportion was similar to that reported in the previous study [4], use of SGLT2is was reported to be higher in this study, possibly reflecting that they are an emerging treatment option in Japan for patients with CKD. As per the multi-country data, the most frequent therapeutic classes used by patients were ACEi or ARB (70%), corticosteroids (49%), and other non-steroidal immunosuppressants (35%; Supplementary Fig. 3). In comparison to multi-country data, corticosteroid use was found to be higher in Japanese patients with C3G. A similar tendency was observed for IgA nephropathy in a previous report that showed a greater use of corticosteroids in Japan than in Europe [21].

The study had a few limitations, so the data should be interpreted with cautious. The primary limitation is the small sample size (only 36 patients from 16 nephrologists), a common challenge when conducting research on rare diseases such as C3 glomerulopathy. While our findings offer important insights into this condition, the limited number of patients prevents us from drawing definitive conclusions or generalizing our results to the wider C3 glomerulopathy population. Further research with a larger cohort will be essential to validate these findings and provide a more comprehensive understanding of the disease. Since the status at diagnosis was answered in the survey, there was a possibility of recall bias for symptoms at diagnosis and potential bias in selection of patients by the physicians. Furthermore, we could not substantiate the patient-reported outcomes in the Japanese population with C3G due to the small number of patients who answered the questionnaire. The survey questionnaires were not designed to collect data on dosage of medications such as corticosteroids. The improvement in disease severity after treatment is subjective as per physicians’ discretions, and the scores are not unified among physicians. However, there is scarcity of data on diagnosis and treatment for Japanese C3G population; therefore, the present data should be important to understand the current situation in Japan.

The findings of this study highlight the challenges in diagnosing and managing C3G due to its heterogeneous clinical presentation and the lack of specific treatment options, suggesting the need for early diagnosis and new therapies to address the underlying mechanisms of C3G. The data collected from nephrologists offer valuable insights into the demographics, clinical characteristics, and treatment approaches for patients with C3G, emphasizing the importance of early diagnosis and personalized treatment strategies to improve patient outcomes.

## Supplementary Information

Below is the link to the electronic supplementary material.

Supplementary file1 (DOCX 70 KB)

## Data Availability

The data underlying this article were provided by Adelphi Real World under license/with permission. Data will be shared on request to the corresponding author with permission of Adelphi Real World.
