# Supplementary material for: Diagnosis and treatment in adult patients with C3 glomerulopathy in Japan: a real-world survey
Source: Clin Exp Nephrol. 2025 Nov 3;30(2):248–55. doi: 10.1007/s10157-025-02779-5 (PMC12886199; doi:10.1007/s10157-025-02779-5)
Supplement: Supplementary file 1 — Supplementary file1 (DOCX 70 KB) [file 10157_2025_2779_MOESM1_ESM.docx]

**Diagnosis and treatment in adult patients with C3 glomerulopathy in Japan: a real-world survey**

Naoki Nakagawa^1^, Yutaro Kotobuki^2^, Michel Kroes^3^, Shunsuke Eguchi^2^, Toshinaga Tsuji^2^, Alice Simons^4^, Susanna Libby^4^, Raisa Sidhu^3^, Serge Smeets^3^, Kazuma Iekushi^2^

**Affiliations**

^1^Division of Cardiology and Nephrology, Department of Internal Medicine, Asahikawa Medical University, Asahikawa, Japan

^2^Novartis Pharma K.K., Tokyo, Japan

^3^Novartis Pharma AG, Basel, Switzerland

^4^Adelphi Real World, Bollington, UK

**Corresponding author**

Prof. Naoki Nakagawa

Department of Internal Medicine, Asahikawa Medical University, Asahikawa, Japan

Email id: [naka-nao@asahikawa-med.ac.jp](mailto:naka-nao@asahikawa-med.ac.jp)

Contact no: +81-166-68-2442

**Supplementary Figures**

**Supplementary Figure 1: Reasons for delayed diagnosis reported by physicians in Japan compared with multinational data**

**
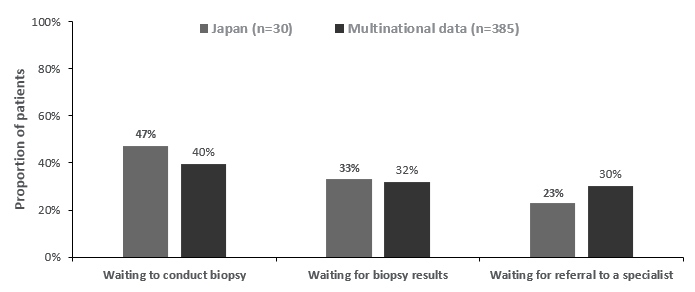
**

**Supplementary Figure 2: a) eGFR and b) proteinuria in Japanese population compared with multinational data**

**a)**


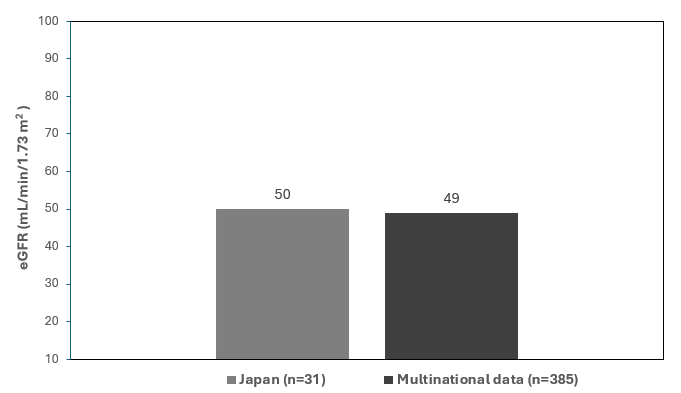


**b)**

**
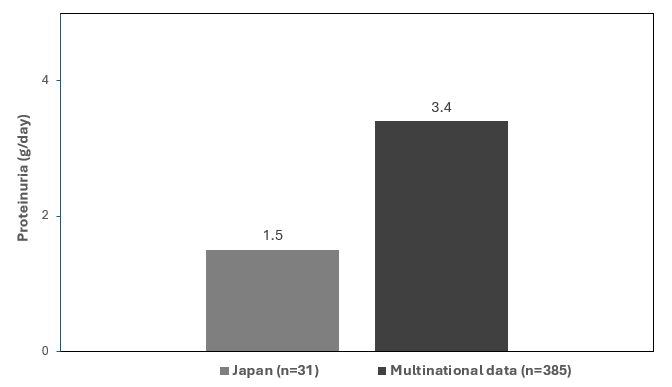
**

**Supplementary Figure 3: Physician-reported treatments in Japanese patients compared with multinational data**

**
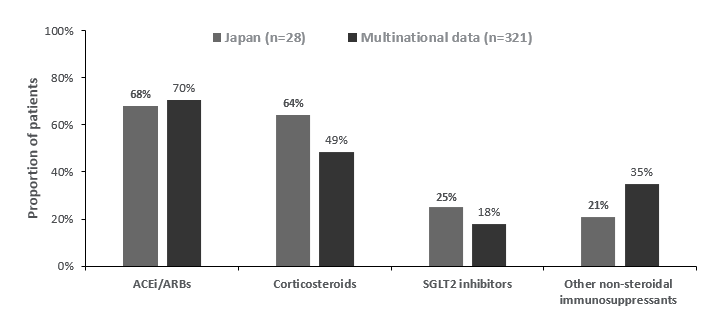
**

ACEi, angiotensin-converting enzyme inhibitors; ARB, angiotensin II receptor blockers; SGLT2, sodium-glucose cotransporter-2.
